# Effects of different supplementary cementitious materials on durability and mechanical properties of cement composite – Comprehensive review

**DOI:** 10.1016/j.heliyon.2023.e17924

**Published:** 2023-07-03

**Authors:** Tsion Amsalu Fode, Yusufu Abeid Chande Jande, Thomas Kivevele

**Affiliations:** a Department of Materials, Energy Science and Engineering, The Nelson Mandela African Institution of Science and Technology, P.O. Box 447, Arusha, Tanzania; b Water Infrastructure and Sustainable Energy Futures (WISE-Futures) Centre of Excellence, The Nelson Mandela African Institution of Science and Technology, P.O. Box 9124, Arusha, Tanzania

**Keywords:** Mechanical property, Cement, Strength, Durability, Natural pozzolana

## Abstract

Ordinary Portland cement is the highest produced cement type in the world, however its production is high energy consumption means expensive, huge natural resource consumptive, and creating high environmental pollution. Hence many researchers studied to reduce the effect of ordinary Portland cement by substituting artificial and natural supplementary cementitious materials (SCMs) commonly in a concrete/mortar mixture. However, the comprehensive effect of different SCMs on various properties of cement composite materials are not well known. So the present study sought to review the effect of different natural and artificial SCMs on the durability and mechanical properties of cement composites, especially due to their doses, types, chemical composition, and physical properties. Hence the review shows that many SCMs used by literatures from different places satisfy ASTM replacement standard based on their chemical compositions. Also, the review indicated as adding 5–20% of different SCMs positively affect mechanical properties, durability, and microstructures of the cement composite materials, specifically as most researchers found isolately adding of 15% SCMs such as bentonite, kaolin, and biomass, 20% addition of volcanic ash and 10% employment of fly ash, silica fume, and zeolite to the cement composites achieves the most optimum compressive and split tensile strength. These observations reveal that most natural pozzolana can more replace cement to give optimum strength, hence can more reduce energy consumption, production cost, and environmental pollution comes due to cement production. Furthermore, most researchers found employing different SCMs generally improves durability, however there is a limited study on the effect of silica fume on water absorption and acidic attack resistance of cementitious materials. Therefore, it is recommended that future research should also focus more to know the effect of silica fume on the durability of cement composites.

## 1 Introduction

Cement is the most fabricated product on Earth by mass and is the second most highly used substance in the world after water [1]. Hence the chemical compositions of cement clinker have to be controlled throughout the process of manufacturing good quality cement, which is mostly affected by the ingredient contents of the cement raw materials [2]. However, manufacturing cement widely contributes to environmental pollution [3,4]. That is because, it is a major consumer of natural resources and the process of cement production requires high temperatures, a large amount of energy consumption, and releasing CO_2_ to the environment [[5], [6], [7], [8], [9]].

Especially the production of ordinary Portland cement is high energy consumption means very expensive, huge natural resource consumption, and creates environmental problems. But, due to the rapid growth of the population in the world, the demand for ordinary Portland cement increasing globally [10,11]. However, every ton of ordinary Portland cement production release about one ton of CO_2_ into the environment [12]. Therefore, researchers have studied the efficiency, availability, and effectiveness of different supplement cementing materials that can be employed as cement replacement which can solve CO_2_ emission comes due to cement production [[12], [13], [14], [15], [16], [17], [18], [19]]. This is mainly because the calcination of SCMs especially clay requires much less calcination temperature than cement clinker [20]. However, there is a gap in which dose and types of SCMs either natural or artificial SCMs most beneficial for cement composites. So the aim of the present study is to review many literatures and indicates the most reliable doses and type of SCMs which can give optimum strength, better microstructure, and more increase durability of the cement composite, hence can highly reduce energy consumption and environmental pollution comes due to cent productions.

### 1.1 Significance of using SCM in cement replacement

Pozzolan is SCM commonly used as cement replacement in the concrete composite for a lot of significance in improving concrete performance, reducing cement consumption with significantly reducing CO_2_ emission [10,16,[21], [22], [23], [24], [25], [26], [27], [28], [29], [30], [31], [32], [33], [34], [35], [36], [37], [38], [39], [40], [41], [42], [43], [44], [45], [46], [47], [48]]. This is more supportive since cement is one of highest produced product and costy in the world due to it requires high energy for production, but adding of SCMs as a cement substitute is highly can reduce the amount of cement consumption required by the construction industry which means indirectly adding of SCMs encourages the economic sustainability of our countries.

Besides to these employing pozzolanic SCMs beneficial in the consumption of calcium hydrate in cement and producing secondary *C*–S–H. These improve the finer pore structure of the cement matrix compared to reference Ordinary Portland cement. Thus the concrete matrix becomes less permeable which can improve concrete durability [47,[49], [50], [51]]. Durability is mostly influenced by the physical and chemical properties of the hardened cement-based materials. Physically, the pore structure, including the pore volume, pore size distribution, tortuosity, and connectivity, which can determine the ease of external gases, liquids, and ions penetrating into the hardened cement matrix, that can deteriorate the concrete lifetime due to the ingress of external matter include carbonation, freeze/thaw damage, sulfate ions ingress [50,52].

In addition to improving durability, employing of SCMs beneficial for lowering cement production costs and environmental impact [18,[52], [53], [54], [55], [56], [57]]. The same findings as SCMs are appealing for the improvement of durability and strength with highly reducing CO_2_ emissions [38,56,[58], [59], [60], [61], [62], [63], [64], [65], [66], [67], [68], [69], [70]]. Also, supplementary cementing materials are an effective means for controlling expansion due to alkali-silica reactions. However, the decomposition of concrete arises when exposed to the sulfuric acid environment, which is the main issue that influences the life cycle, performance, and maintenance costs of concrete structures. Hence employing SCMs in a cement composite can enhance the sulfate resistance of concrete [12,49], improves resistance to chemical corrosion fracture toughness, and can resist low and high temperatures [71].

Additionally, SCMs from by-product or waste reflects promising performance as a partial replacement of cement composite materials which can reduce the consumption of natural resources [72] and reduces the production cost of cement factories with fulfilling demand-supply capacity [73]. Employment of SCMs in cement composite materials can be influenced by the amount of cement which is if the amount of cement is not sufficient, there is no reaction of calcium hydroxide by supplementary materials. And also the fineness of the supplementary cementitious material mostly affects the reactivity of SCM with free lime in cement [74]. That means the employment of SCMs in a cement lower amount of Portland clinker, which produces portlandite at hydration time and SCMs improves the consumption of portlandite in pozzolanic reaction [14,75].

Also, Ivashchyshyn et al. [76] studied the low-emission cement achieved by SCMs especially fly ash and zeolite decease the bleeding because its surface area is higher than cement components with reducing energy consumption and improves strength to produce pozzolanic cement compared to ordinary Portland cement [77]. In addition, ductility is the potential of the structures to undergo much inelastic deformation beyond initial yield deformation that is without significant lessening of strength or stiffness. So the ductile structural membranes expected to withstand overloads, load reversals, impact, and structural moment may be caused by foundation settlement or volume change, hence employing SCMs in cement composite improves ductility [78,79].

Besides these, concrete is alkaline and chemical attack resistant, though can be affected by the acid attack in liquids having pH value below 6.5 and severe at below 5.5 and very severe below 4.5 pH value, that is because of easy dissolution and leaching of calcium hydroxide in hardened concrete, so SCMs is beneficial by its micro filling ability that can protect the entrance of acids from the reduction of concrete durability [50,80,81].

Furthermore, the incorporation of industrial by-products such as slag, fly ash, and silica fume positively affects concrete mixture [12,81]. Cement quality is typically assessed by the development of the compressive strength of mortar or concrete [2]. Also, long-term enhancement of compressive strength is evidence of hydraulic reaction and pozzolancity of the SCMs [50,82]. Generally, SCMs either industrial by-products such as fly ash, silica fume, or natural Pozzolan mean like bentonite, kaolin, zeolite, and volcanic ashes are appealing for the technical, economical, and environmental benefit that is of great importance for sustainable building construction [9,64,83,84]. Therefore, the present study is aimed to know the range of most positive reflections of SCMs on physical, mechanical, durability, and microstructural properties of cement composite materials due to its doses, types, and chemical reactions through compressively reviewing different literature.

### 1.2 Chemical composition of supplementary cementitious materials

#### 1.2.1 Chemical composition of natural supplementary cementitious materials

The pozzolanic reaction of natural SCMs mainly depends on their chemical composition, chemical reactivity index, and mineralogical composition [6]. Also, Setina et al. [85] reported as pozzolanic activity of pozzolana is strongly dependent on their chemical composition mostly on the amounts of reactive silica. As presented in Table 1, all reviewed natural SCMs satisfy ASTM C618 [86] requirements, by the addition of the values of sulfur dioxide (SiO_2_), Aluminum oxide (Al_2_O_3_), and Iron oxide (Fe_2_O_3_) greater than 70% for natural pozzolanic materials added to concrete manufacturing. This is due to the reason SCMs are used to reduce the production cost of cement and environmental pollution, but as well those three oxides have the potential to actively react with free lime from clinker to produce calcium silicate hydrate (*C*–S–H) and calcium aluminate silicate hydrates (*C*-A-*S*-H) which is the crucial compound that improve strength, durability and make dense microstructure of cement composite materials. In addition to that increasing the sum of SiO_2_, Al_2_O_3_ and Fe_2_O_3_ oxides increases the strength, mainly increasing of strength caused by SiO_2_. That is because SiO_2_ is the most important oxide which can improve the pozzolanic reaction in pozzolana [87].

**Table 1:** Chemical composition of natural supplementary cementitious materials used in various research.

Bentonite													
SiO_2_	Al_2_O_3_	Fe_2_O_3_	CaO	MgO	SO_3_	Na_2_O	K_2_O	TiO_2_	P_2_O_5_	Cl	LOI	SAF	References
48.80	15.54	6.44	5.22	3.50	–	2.19	0.75	0.49	0.13	–	15.73	70.78	[88]
49.17	14.55	7.37	0.75	2.23	–	3.25	0.68	0.22	–	–	–	71.09	[89]
64.25	15.60	4.60	1.63	2.50	0.00	2.80	3.86	0.60	0.21	–	0.24	84.45	[90]
49.17	14.55	7.37	0.75	2.23	–	3.25	0.68	0.22	–	–	–	71.09	[89]
70.70	13.80	1.49	2.30	2.26	0.29	2.56	0.33	0.20	0.05	0.00	5.12	85.99	[91]
64.23	19.39	5.19	3.79	1.81	0.08	1.49	1.52	0.09	0.02	–	1.12	88.81	[92]
67.03	16.31	1.76	2.81	7.15	0.05	3.10	1.43	0.13	0.09	0.04	–	85.10	[93]
52.80	16.40	5.80	4.60	1.40	–	0.62	0.70	–	–	–	9.6	75.00	[94]
57.98	19.70	12.46	1.11	1.71	–	1.25	1.51	1.62	–	–	–	90.14	[95]
52.80	16.40	5.80	4.60	1.40	–	0.62	0.70	–	–	–	9.60	75.00	[96]
56.13	21.20	3.23	3.16	4.32	–	4.14	1.01	0.55	0.12	–	6.11	80.56	[97]
76.17	14.38	3.17	1.88	1.11	0.91	1.06	0.54	0.60	0.04	–	4.86	93.72	[98]
**Kaolin**													
71.78	14.04	5.50	0.46	–	0.00	–	4.58	0.33	0.21	–	0.72	91.32	[99]
56.30	37.42	1.20	0.02	0.46	0.08	0.07	3.51	0.69	–	–	–	94.92	[100]
66.52	21.96	0.85	0.20	0.37	–	0.83	0.00	–	–	–	–	89.33	[46]
74.2	17.60	0.85	3.32	0.25	0.43	0.00	0.34	–	–	–	–	92.65	[101]
62.18	21.67	6.05	3.01	3.41	–	1.03	1.85	1.03	–	–	0.50	89.90	[102]
55.10	34.10	5.40	0.28	0.25	0.01	0.10	0.02	2.00	1.00	–	1.50	94.60	[103]
50.62	46.91	0.38	0.02	0.09	0.08	0.28	0.18	1.29	–	–	0.00	97.91	[104]
57.37	38.63	0.77	0.03	0.07	0.15	0.39	0.49	–	0.61	–	1.04	96.77	[105]
48.45	34.75	1.28	0.03	0.85	–	0.02	2.40	0.85	0.09	–	–	84.48	[106]
58.52	35.54	1.15	1.24	0.19	0.06	0.25	0.05	0.04	0.09	–	2.74	95.21	[107]
63.51	29.68	1.22	0.48	0.48	0.30	0.14	1.79	0.66	–	–	1.95	94.41	[108]
58.16	37.63	0.79	0.17	0.19	0.35	0.09	0.08	1.13	0.41	–	0.93	96.58	[109]
54.3	38.3	4.28	0.39	0.08	–	–	0.50	–	–	–	0.68	96.88	[110]
**Volcanic ash**													
47.40	18.52	10.04	7.90	6.04	0.34	2.58	1.07	1.62	0.64	0.01	2.21	75.96	[111]
40.48	12.90	17.62	11.83	8.33	–	3.60	1.67	–	1.37	–	1.60	71.00	[112]
73.68	14.69	2.63	2.02	0.28	0.03	2.27	3.88	–	–	0.06	2.39	91.00	[113]
44.95	17.32	9.49	12.36	4.20	0.01	3.00	1.39			0.00	6.72	71.76	[114]
46.48	14.74	12.16	8.78	8.73	–	3.39	1.27	2.31	0.63	–	1.32	73.38	[115]
47.40	18.56	10.04	7.90	6.04	0.34	2.58	1.07	1,62	0.64	0.01	2.21	76.00	[116]
46.96	17.81	9.74	10.97	2.46	0.84	3.29	1.57	–	–	–	–	74.51	[117]
65.20	14.9	3.50	3.20	0.60	0.00	3.80	3.70	0.70	–	–	3.90	83.60	[118]
46.50	19.28	11.22	8.50	5.48	0.14	2.70	3.61	1.88			0.66	77.00	[119]
44.95	16.91	9.47	14.59	3.70	0.20	1.34	1.35	–	–	–	4.30	71.33	[120]
47.40	18.57	10.04	7.90	6.06	0.34	2.58	1.07	1.62	0.64	0.01	2.21	76.01	[121]
**Zeolite**													
68.95	11.14	0.94	4.83	0.79	0.07	0.95	0.90	–	–	–	–	81.03	[122]
63.72	11.40	2.73	3.29	0.05	0.13	1.02	2.83	0.29	0.03	–	14.20	77.85	[123]
75.34	8.77	1.30	4.60	0.55	0.05	1.22	2.41	–	–	–	–	85.41	[124]
63.32	11.70	0.32	3.60	1.20	0.09	–	–	–	–	–	8.49	75.34	[125]
64.70	11.21	1.38	2.08	0.79	0.03	–	3.78	–	–	–	8.00	77.29	[126]
63.32	11.70	0.32	3.60	1.20	0.09	–	–	–	–	–	4.49	75.34	[127]
68.80	10.70	0.72	2.54	0.83	0.22	1.55	1.44	–	–	–	12.78	80.22	[128]
62.78	12.20	2.37	5.09	2.65	0.01	0.42	0.74	–	0.05	0.05	12.36	77.35	[11]
67.44	10.80	0.84	1.24	0.33	0.47	3.71	0.19	–	–	–	–	79.08	[129]
69.20	15.28	3.01	2.24	1.40	0.45	2.20	2.10	–	–	–	4.12	87.49	[130]
67.79	13.66	1.44	1.68	1.20	0.50	2.04	1.42	–	–	–	10.23	82.89	[131]

#### 1.2.2 Chemical composition of artificial supplementary cementitious materials

As presented in Table 2, all reviewed artificial supplementary cementitious materials satisfy ASTM C618 [86] requirements of addition for the values of sulfuric dioxide (SiO_2_), Aluminum oxide (Al_2_O_3_), and Iron oxide (Fe_2_O_3_) greater than 70% for pozzolanic materials added to concrete manufacturing and also can see as the summation of three oxides SiO_2_, Al_2_O_3_, and Fe_2_O_3_ of most of the artificial supplementary cementitious materials are more greater than the natural SMCs, that is also known as the summation of those three oxides can significantly play important role in the pozzolanic reaction. Hence increasing the sum of those three oxides significantly increases the strength and durability of cementitious materials [87].

**Table 2:** Chemical composition of manmade supplementary cementitious materials used in various research.

Rise Ash/Biomass Ashes													
SiO_2_	Al_2_O_3_	Fe_2_O_3_	CaO	MgO	SO_3_	Na_2_O	K_2_O	TiO_2_	P_2_O_5_	Cl	LOI	SAF	References
62.00	31.50	1.79	0.48	0.39	–	–	–	–	–	–	0.71	95.29	[132]
88.84	0.80	0.39	1.78	0.92	0.35	1.10	2.80	0.04	0.61	–	2.02	90.03	[133]
90.60	1.70	0.70	0.10	0.80	–	–	2.40	–	–	–	6.00	93.00	[134]
83.10	2.15	1.10	4.70	1.50	1.20	0.10	2.96	–	2.05	–	1.13	86.35	[135]
89.91	0.13	0.95	2.75	0.30	0.83	0.01	2.75	–	–	–	2.99	90.99	[136]
92.80	0.15	0.17	0.70	0.77	–	0.08	3.35	–	1.07	–	–	93.12	[137]
91.15	0.41	0.21	0.41	0.45	0.62	0.05	6.25	–	–	–	0.45	91.77	[138]
86.90	0.24	0.10	1.03	0.34	0.14	0.11	2.11	–	–	–	1.53	87.24	[139]
66.12	14.99	7.16	2.57	1.19	0.26	0.54	3.52	1.13	1.14	–	9.34	88.27	[140]
78.34	8.55	3.61	2.15	–	–	0.12	3.46	0.50	1.07	–	–	90.50	[141]
77.25	6.37	4.21	4.05	2.61	0.11	1.38	2.34	0.58	0.59	–	–	87.83	[142]
71.11	4.85	6.80	4.04	–	–	2.25	3.89	0.40	0.39	–	–	82.76	[143]
**Silica fume**													
90.36	0.71	1.31	0.45	–	0.41	0.45	1.52	–	–	–	3.11	92.38	[144]
92.50	0.72	0.96	0.48	1.78	–	0.50	0.84	–	–	–	1.55	94.18	[145]
94.17	1.10	1.45	1.20	0.18	0.25	0.45	1.20	–	–	–	–	96.72	[146]
96.09	0.14	0.07	0.39	0.12	0.09	0.19	0.42	–	–	–	2.50	96.30	[147]
96.40	0.25	0.45	0.25	0.45	0.15	0.20	0.50	–	–	–	–	97.10	[148]
91.80	0.54	1.92	0.70	0.49	0.35	0.68	1.20	–	–	–	1.67	94.26	[149]
91.57	0.38	0.15	0.32	4.05	–	0.55	2.58	–	–	–	1.68	92.10	[150]
99.80	0.11	0.09	0.40	0.20	–	0.20	0.50	–	–	–	3.50	100	[151]
90.21	0.12	0.15	0.30	0.73	0.01	0.46	1.51	–	–	–	–	90.48	[152]
85.04	0.97	1.04	1.63	1.20	1.22	0.48	0.36	0.21	–	–	2.03	87.05	[153]
93.60	1.32	0.37	0.49	0.97	0.10	0.31	1.01	–	–	–	–	95.29	[122]
90.82	0.47	2.26	0.52	1.55	0.68	1.02	1.95	–	0.12	0.22	–	93.55	[154]
93.60	1.32	0.37	0.49	0.97	0.10	0.31	1.01	–	–	–	–	95.29	[155]
93.67	0.83	1.30	0.31	0.84	0.16	0.40	1.10	–	–	–	2.10	95.80	[156]
**Fly ash**													
51.59	23.78	6.58	4.30	0.49	3.05	1.09	3.05	1.03	–	–	–	81.95	[157]
55.23	25.95	10.17	1.32	0.31	0.18	1.59	1.59	–	–	–	5.25	91.35	[158]
48.22	29.27	1.19	9.60	2.56	–	1.84	1.14	0.26	0.18	–	5.78	78.68	[97]
55.40	20.76	11.26	3.88	1.38	–	0.84	2.09	–	–	–	–	87.42	[159]
52.90	33.20	5.23	2.92	1.06	0.73	0.72	1.20	1.17	–	–	–	91.33	[160]
57.20	28.81	3.67	5.16	1.48	0.10	0.08	0.94	–	–	–	–	89.68	[161]
55.27	26.72	6.66	2.35	0.81	0.47	–	3.01	1.89	1.95	–	3.20	88.65	[162]
50.96	25.88	8.25	2.15	2.60	0.65	1.26	2.65	1.36	0.35	–	3.20	85.09	[84]
51.45	26.00	7.65	3.58	1.71	0.93	0.10	3.84	–	–	–	4.85	85.10	[163]
54.20	27.17	6.23	6.89	1.16	0.11	0.17	0.67	1.79	0.97	0.01	3.65	87.60	[164]
54.70	29.00	6.74	1.29	0.80	0.10	1.88	–	–	–	–	2.72	90.44	[165]
57.80	26.30	6.20	1.60	0.80	0.30	–	3.00	1.30	0.10	–	–	90.30	[166]
53.01	27.89	8.71	4.23	1.84	0.96	0.58	1.63	–	–	–	1.15	89.61	[167]
57.01	20.96	4.15	9.79	1.75	–	2.23	1.53	0.68	–	–	1.25	82.12	[168]

## 2 Natural supplementary cementitious materials

Natural SCMs are natural pozzolana from the natural sedimentation of volcanic ash or lava that involves active silica, used as cementitious materials when combined with free lime [169,170]. Hence using of SCMs of natural pozzolana in concrete mixtures contribute a lot of significance in the fresh and hardened properties of concrete, enhancing workability, reducing the heat of hydration, lower permeability, improve ultimate strength and durability [[171], [172], [173], [174], [175], [176], [177], [178], [179], [180], [181], [182], [183], [184], [185]]. Also, increasing the content of natural pozzolana in concrete increases the chloride migration coefficient [186], increases sulfuric acid resistant [120,[187], [188], [189]], and decreases permeability & water absorption which can improve the long-term strength gain of concrete [90,96,[190], [191], [192], [193], [194], [195], [196]].

### 2.1 Bentonite

Bentonite is alumina-siliceous material [97], mainly plastic clay consisting of 87% earth minerals with montmorillonite and fulfill pozzolanic properties. There are two types of bentonite namely, swelling sort/sodium bentonite and non-swelling sort/calcium bentonite [176,197]. Using bentonite in a concrete mixture reduces the contents of portlandite which is mainly due to the hydration reaction between cement and bentonite. In most cases, adding bentonite in concrete by replacing cement content have the ability to consume more portlandite compared with kaolin replacement in a concrete mixture [198]. Hence, employing bentonite in cementing material improve strength and durability by enhancing the resistance to acidic attack of cement matrix [[199], [200], [201], [202], [203], [204], [205], [206]]. That is more presented in Table 3 as many researchers found optimum strength by using 5–15% of bentonite as a cement substitute in cement composite. Also mixing bentonite in concrete, improve concrete bleeding and cohesiveness of concrete in low-intensity level [207].

**Table 3:** Effect of bentonite on some of the mechanical properties and durability recorded by different researchers.

Dose range	W/C	Curing time	Optimum compressive strength		Optimum split tensile strength		Optimum flexural strength		Durability of adding bentonite comparing with control mixtures		References
			Dose (%)	Age (days)	Dose (%)	Age (days)	Dose (%)	Age (days)	Water absorption	Acidic attack	
0,5,10,15,20,25	0.40	0,7,28,56	15	7,28, 56	15	7,28, 56	–	–	Increase	Decrease	[208]
0,10,15,20,25,30,35	0.5	7,28,180	0	0,10, 15,20, 25,30, 35	15	7,28, 180	–	–	–	–	[200]
0,5,10,15,20	0.48	7,28	5	7,28	5	7,28	5	7,28	–	–	[209]
0,5,10,15,20	0.47	7,28	5	7,28	5	7,28	5	7,28	–	–	[210]
0,5,10,15,20	0.50	28,56,90	15	28,56,90	15	28,56,90	15	28,56,90	–	Decrease	[204]
0,10,15,20	–	28	15	28	15	28	–	–	–	–	[211]
0,5,10,15,20	0.53	0,3,28,90	15	90	15	90	–	–	Decrease	Decrease	[96]
0,5,10,15,20	0.40	0,7,28,56,90	15	56,90	–	–	–	–	Increase	–	[212]
0,5,10,15	0.40	0,3,7,28	10	28	10	28	10	28	–	–	[213]
0,3,6,9,12,15,18,21	0.58	3,7,28,56	15	56	–	–	–	–	Decrease	Decrease	[214]

**Fig. 1:**
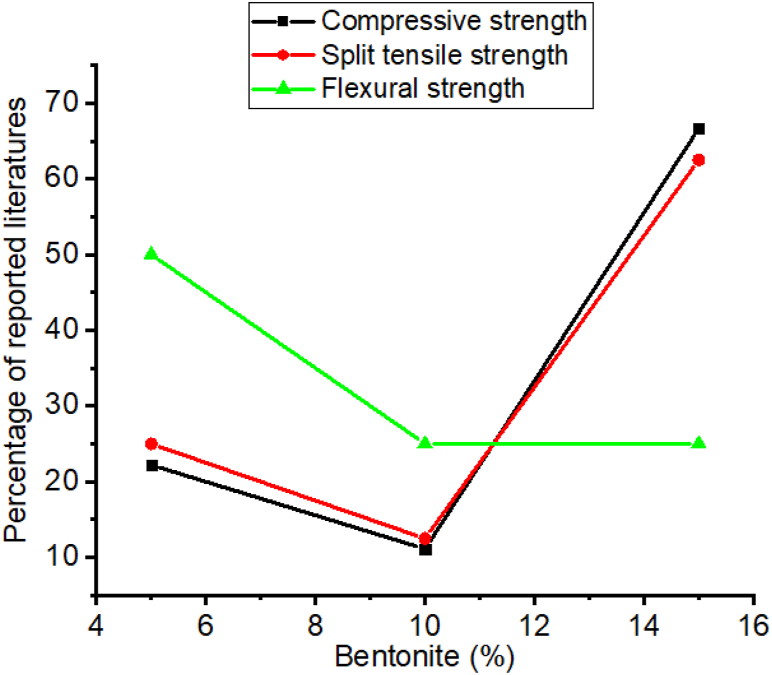
Summary from Table-3 that most researchers reported on the influence of bentonite doses for optimum compressive strength, split tensile strength, and flexural strength.

**Fig. 2:**
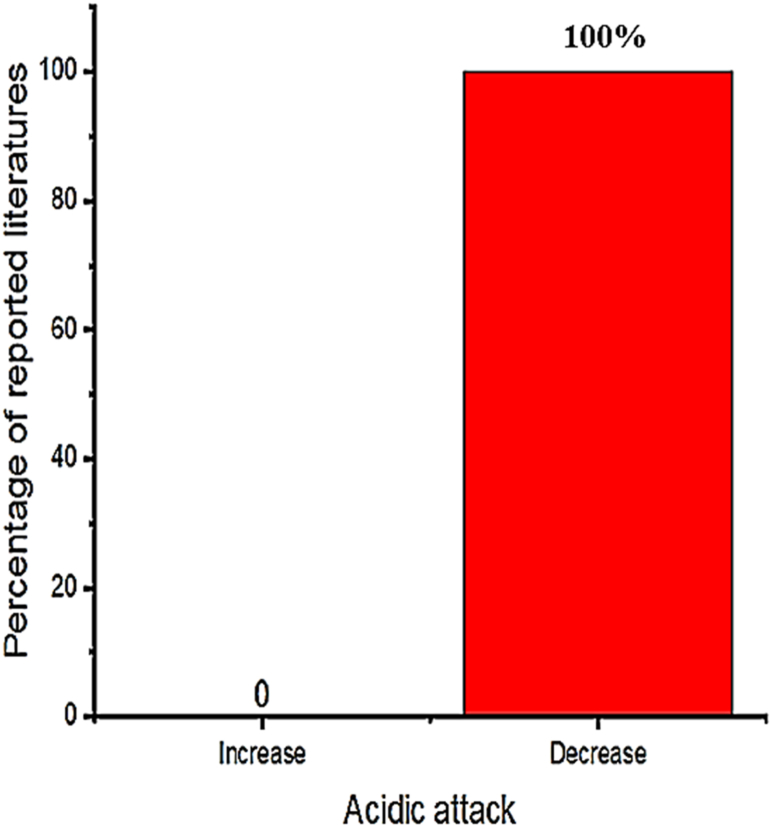
Summary from Table-3 that most researchers reported on the influence of bentonite on acidic attack.

Employing bentonite improves the strength and durability of concrete in construction works [174,204,209,210,215]. Specifically incorporating bentonite in concrete increases compressive strength compared to the control mixture [212,216,217]. That is as presented in Fig. 1 most researchers reported the incorporation of bentonite in cementitious material significantly enhances strength, specifically using 15% of bentonite replacement can highly improve compressive and splitting tensile strength, also 5% of bentonite addition highly improves flexural strength. Besides these, Fig. 2 shows the inclusion of bentonite in cementitious materials reduces acidic attack by enhancing the resistance of chloride penetration and sulfate resistance compared to reference concrete without bentonite [96,204,218,219]. This is mainly due to the amorphous silicate matrix actively reacts with Portlandite to form secondary *C*–S–H gel that improves the microstructure and strength of the final hydrated cement matrix, which is mostly dependent on the hydration reaction of pozzolana and cement phase [107,[220], [221], [222], [223], [224]].

**Fig. 3:**
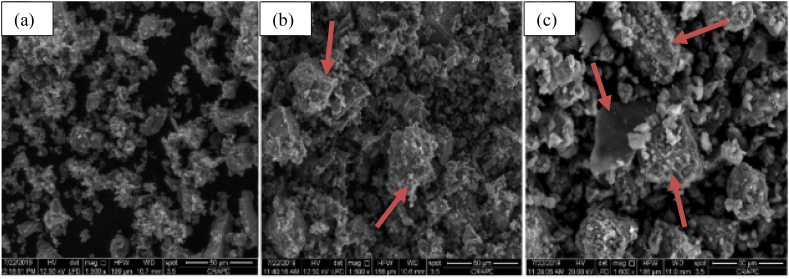
Microstructure of cement composite materials (a) with OPC, (b) and (c) OPC with bentonite by [227].

In addition to these, the mixture of concrete that incorporates bentonite reflects a much denser microstructure compared to the reference mixture [225]. As shown in Fig. 3a-c, cementitious composite materials without bentonite have small particles having many pores between each other; however, the samples with SCMs of bentonite have large-sized particles, more dense, and very low pores between the particles. This means, employing bentonite significantly forms a dense structure and lower porosity compared to without bentonite. That is because of the dissolution of montmorillonite and the production of secondary minerals *C*–S–H gel in concrete containing bentonite [226].

### 2.2 Kaolin

**Fig. 4:**
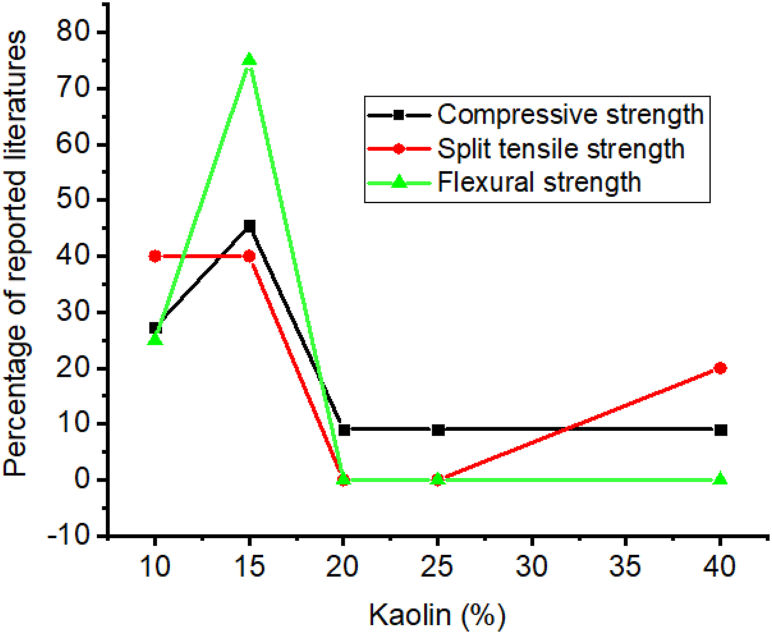
Summary from Table-4 that most researchers reported on the influence of kaolin doses for optimum compressive strength, split tensile strength, and flexural strength.

Kaolin is natural pozzolana require calcination to form reactive pozzolana at a temperature between 700 and 1200 °C. So calcined kaolin improves the strength, and durability of Portland cement concrete and other cement composite materials [8,101,180,193,[228], [229], [230]]. As presented in Table 4 most researchers employed 10–25% of kaolin in cementitious materials to improve strength, specifically as shown in Fig. 4 most of the literature reported adding 15% by mass of cement can radically increase compressive strength, split tensile strength, and flexural strength. This is because of two actions, firstly pore filling ability of kaolin and secondly through the active pozzolanic reaction of kaolin with calcium hydroxide which can form an extra crystalline nucleus that can refine the hydrated gel structures [231].

**Table 4:** Effect of kaolin on some of the mechanical properties and durability recorded by different researchers.

Dose range	W/C	Curing time	Optimum compressive strength		Optimum split tensile strength		Optimum flexural strength		Durability of adding kaolin comparing with control mixtures		References
			Dose (%)	Age (days)	Dose (%)	Age (days)	Dose (%)	Age (days)	Water absorption	Acidic attack	
0,10,20,30,40,50	–	7,14,28	10	7,14,28	10	7,28	10	7,28	–	–	[232]
0,2.5,5,7.5,10	0.35	7,14,28	10	7,14,28	–	–	–	–	–	–	[233]
0.5,10,15,20	0.32	7,28	15	7,28	15	28	15	28	–	–	[110]
0.5,10,15,20	0.37	3,28,90	15	28,90	–	–	0	90	Decrease	–	[234]
0.5,10,15,20	0.32	7,28	15	7,28	15	28	15	28	–	–	[110]
0,5,10,15,20,25,30	0.45	3,7,28	25	3,7,28	–	–	–	–	–	Decrease	[105]
3,5,10	0.53	7,28,60,90	10	7,28,60,90	10	7,28, 60,90	–	–	Decrease	–	[61]
0.10,20,30	0.50	2,28,90	20	2,28,90	–	–	–	–	–	–	[235]
0,10,20,30,40,50	0.50	7,56,90,500	40	7,56,90,500	40	7,56, 90,500	–	–	–	–	[107]
0,5,10,15	0.50	7,28	15	7,28	–	–	15	7,28	–	–	[193]
0,5,10,15,20,25	0.55	7,28	15	7,28	–	–	–	–	Decrease	–	[102]
0,30	0.50	2,7,28, 90	0	2,7,28, 90	–	–	–	–	–	Decrease	[236]

**Fig. 5:**
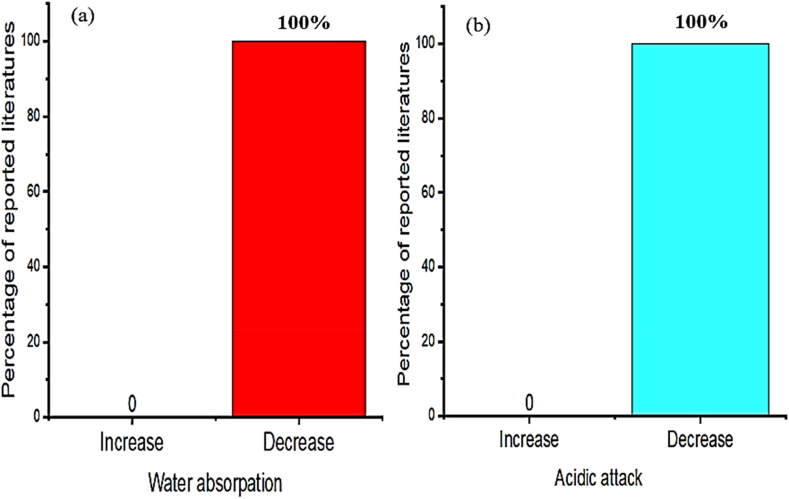
Summary from Table-4 that most researchers reported on the influence of kaolin on (a) water absorption and (b) acidic attack.

As shown in Fig. 5a–b employment of kaolin in cementitious materials reduce water absorption and reduce acidic attack by filling an air-void matrix. This is due to the kaolin micro-filling effect of the pores of cement composite materials that reduce the migration of water or acids to the matrix. Also, Karahan et al. [237] reported as increasing meta-kaolin doses reduces porosity and water absorption. This is because, the pozzolanic reaction that can alter the microstructure of concrete with the chemistry of hydration reaction by consuming free lime and instead produces secondary calcium silicate hydrate (*C*–S–H) which can improve strength and durability [195], and also by its filler and pozzolanic effect [238]. Similar observation with M. A. Elahi et al. [49] as the inclusion of kaolin in cement composite enhances the sulfate resistance, which is by replacing kaolin from 5 to 25% to a mass of cement. Besides these, employing kaolin in cementitious materials improves chloride ion penetration due to the positive effect of kaolin in the reduction of porosity and water [236].

**Fig. 6:**
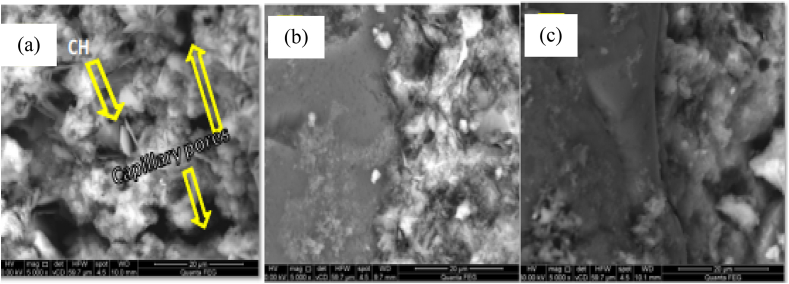
Microstructure of cement composite materials (a) with OPC, (b) and (c) OPC with kaolin by Ref. [239] permission Elsevier.

As presented in Fig. 6a the microstructure of plane cementitious materials has a nonuniform arrangement, high capillary pores, and also there is a high micro gap between cement paste and aggregates, that is maybe because of the collection of free lime crystal in the transition zone of interfacial [239]. As shown in Fig. 6b–c, the image of the cement composite sample without kaolin reflects not agglomerated particles having big and many pores, however in the samples of cementitious composite materials with kaolin can see dense and compacted structures having low pores. Hence replacement of kaolin to cement composite material reveals a denser microstructure with low porosity compared to the plane cementing material. This is due to the micro-filling ability of ultrafine kaolin particles which can make dense microstructures and fill the pore that exists in between aggregates and cement paste.

### 2.3 Volcanic ash

Volcanic ash is natural pozzolanic material, that occurred during volcanic eruptions when magma or molten rock and solid rock shatter separated form fine particles of clay to the size of sand [240]. As presented in Table 5 employment of 5–20% volcanic ash improves the mechanical properties and durability of cementing materials. That is due to volcanic ash actively reacting with free lime (calcium hydroxide) and due to the hydration of cement, which is amorphous silica reacts in volcanic ash react with lime and form cementitious material *C*–S–H gel, it is crucial for the improvement of concrete durability, enhances strength, and lessen the rate of heat liberation important for mass concrete [241]. Also adding volcanic ash to cement composite enhances workability, reduces porosity [111], and significantly reduces concrete/cement composite production costs [242,243]. Generally, from the observation of many literatures, partial substitution from 5 to 20% of volcanic ash is beneficial for the improvement of workability, compressive strength, and durability in addition to making economical and environmentally friendly cement composite materials, which is mainly due to its high pozzolanic reactivity with portlandite in a cement.

**Table 5:** Effect of volcanic ash on some of the mechanical properties and durability recorded by different researchers.

Dose range	W/C	Curing time	Optimum compressive strength		Optimum split tensile strength		Optimum flexural strength		Durability of adding volcanic ash comparing with control mixtures		References
			Dose (%)	Age (days)	Dose (%)	Age (days)	Dose (%)	Age (days)	Water absorption	Acidic attack	
0,5,10, 15	0.30	28,56	5	28,56	–	–	–	–	–	–	[241]
0,10,20,30	0.48	7,28,90	10	90	–	–	–	–	Decreases	–	[111]
0,20	0.40	7,28,90	20	90	–	–	–	–	–	–	[244]
0,15,30	0.38	1,7,28,56,90,180	15	90,180	–	–	–	–	Increases	–	[245]
0,10,20,30	0.45	7,14,21, 28	20	21,28	–	–	–	–	–	–	[246]
0,10,20,30,40, 50	0.35	28	20	28	–	–	–	–	–	–	[243]
0,10,20,30	0.48	7,28,91	30	28,90	–	–	–	–	–	–	[247]
0,5,10, 15,20	0.35	7,28,120	5	7,28, 120	–	–	–	–	–	–	[248]
0,10,20,30	_	1,7,14,28,90,180	10	90,180	10	28,90,180	10	28,90,180	Decreases	–	[116]

**Fig. 7:**
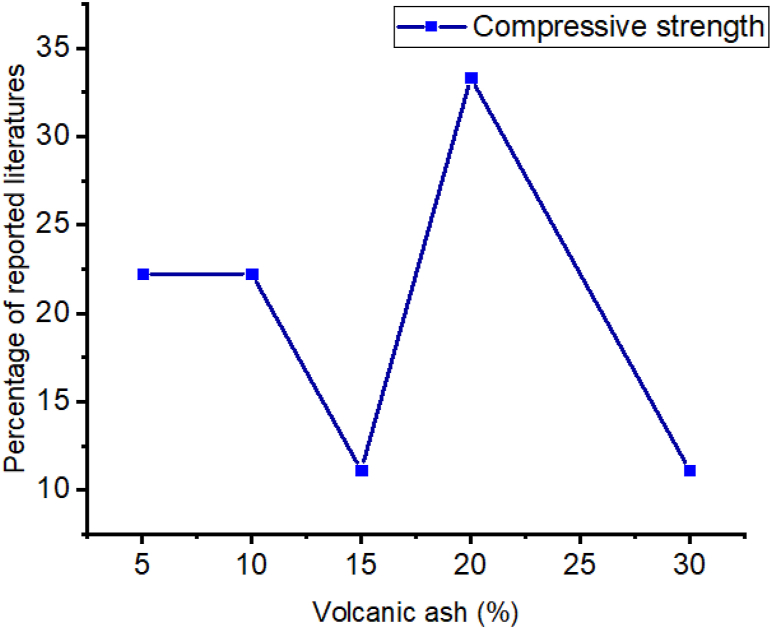
Summary from Table-5 that most researchers reported on the influence of volcanic ash doses for optimum compressive strength.

**Fig. 8:**
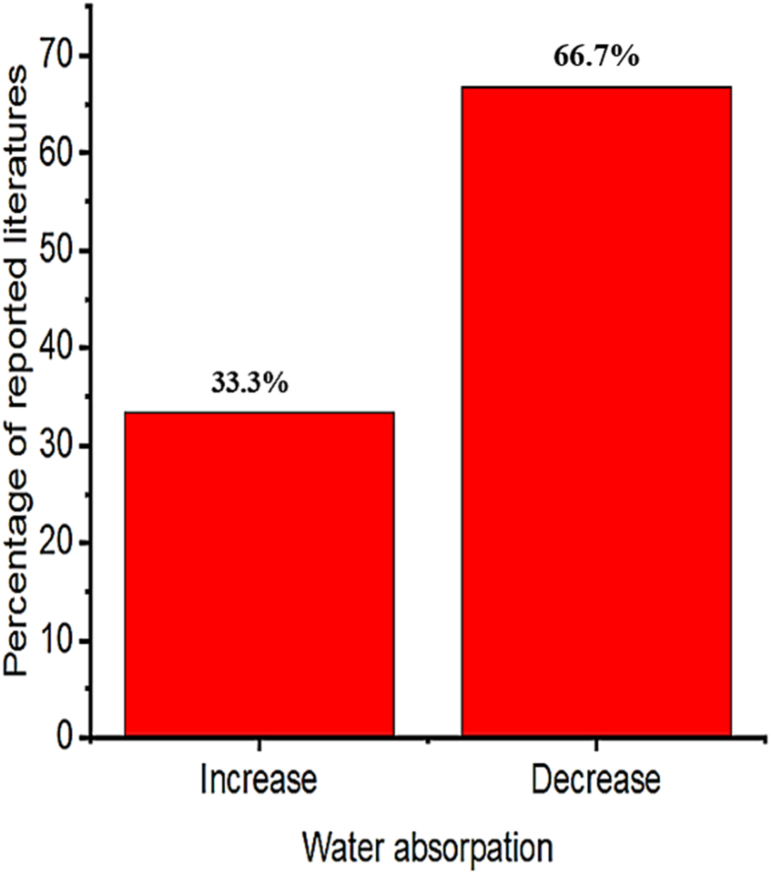
Summary from Table-5 that most researchers reported on the influence of volcanic ash on water absorption.

The employment of volcanic ash actively reacts in cement hydration reactions to form secondary *C*–S–H gel which significantly improves strength [246]. This is more shown in Fig. 7 as most of the literature found the optimum compressive strength at 20% addition of volcanic ash to cement composites. Also as presented in Fig. 8 many researchers reported as the employment of volcanic ash reduces water absorption. That is because the use of volcanic ash in cement composite increase the densification of cement slurries that protects the penetration of water [246,249].

### 2.4 Zeolite

Zeolite can be used in lime mortar and concrete which is formed from a change of volcanic ash, tuff, and others. It is used in many cement industries over the world as a clinker partial substituent. Most of the effectiveness of zeolite in cementitious material significantly can reduce chloride ion penetration to concrete matrix [80,127]. This is mainly due to the interlocking and micro-filling ability of zeolite particles through having higher surface area and active pozzolanic reaction with free lime that can lessen the capillary pores in concrete/mortar matrix, hence reducing the penetration of chloride ions.

**Fig. 9:**
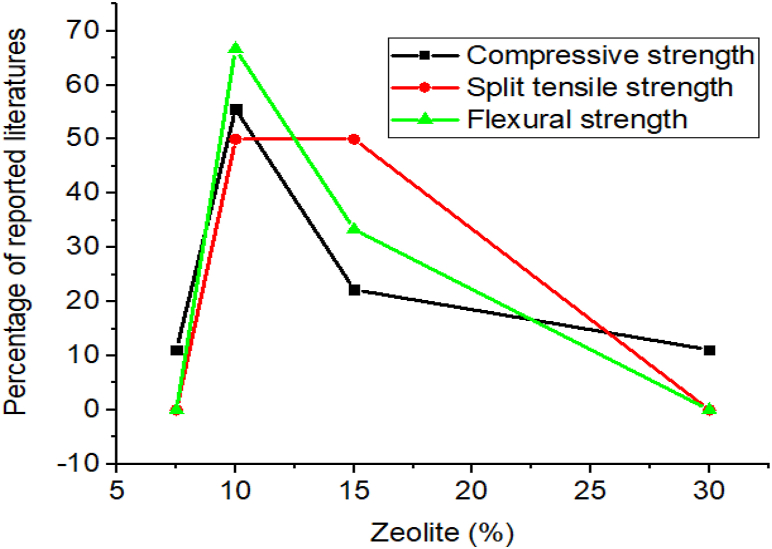
Summary from Table-6 that most researchers reported on the influence of zeolite doses for optimum compressive strength, split tensile strength, and flexural strength.

Also, the inclusion of zeolite in cementitious materials improves concrete mechanical properties, especially compressive strength, and durability [123,126,[250], [251], [252]]. As presented in Table 6 using 7.5–30% of zeolite in cementing materials highly enhance strength and durability, however as shown in Fig. 9, many researchers found employing 10% of zeolite in cementitious materials can give optimum compressive strength, split tensile strength, and flexural strength. That is because zeolite actively reacts with free calcium hydroxide to form *C*-A-*S*-H and *C*–S–H which are crucial compounds to improve the mechanical properties of concrete [11,126]. Also, Sicakova et al. [253] studied the effect of blinding zeolite on the long-term properties of cement-based composites for building material and found as incorporating zeolite in concrete increase the long-term compressive strength (three years) and density of concrete compared to control concrete mix.

**Table 6:** Effect of zeolite on some of the mechanical properties and durability recorded by different researchers.

Dose range	W/C	Curing time	Optimum compressive strength		Optimum split tensile strength		Optimum flexural strength		Durability of adding Zeolite comparing with control mixtures		References
			Dose (%)	Age (days)	Dose (%)	Age (days)	Dose (%)	Age (days)	Water absorption	Acidic attack	
0,10,20,30,40	0.35	42	10	42	–	–	–	–	–	–	[254]
0,10,20,30	0.40	7,28	10	7,28	–	–	–	–	–	Decrease	[255]
0,10,15	0.35,0.40,0.45,0.50	7,28, 90,270	10	7,28, 90,270	10	90	–	–	Decrease	Decrease	[256]
0,2.5,5,7.5,10	0.45	7,28	10	7,28	–	–	–	–	Decrease	–	[194]
0,5,10, 15,20	0.50	3,7,28,90	15	28,90	–	–	15	28,90	Decrease	Decrease	[127]
0,10,20,30	0.40	2,7,28,60,90,180	10	180	–	–	10	180	Increase	–	[11]
0,5,10, 15	0.48	28	15	28	15	28	10	28	–	–	[131]
0,30,40	0.30	28,56	30	56	0	28,56	–	–	–	Decrease	[80]
0,7.5,15,22.5,30	0.38	7,28, 90	7.5	90	–	–	–	–	Increase	–	[257]

**Fig. 10:**
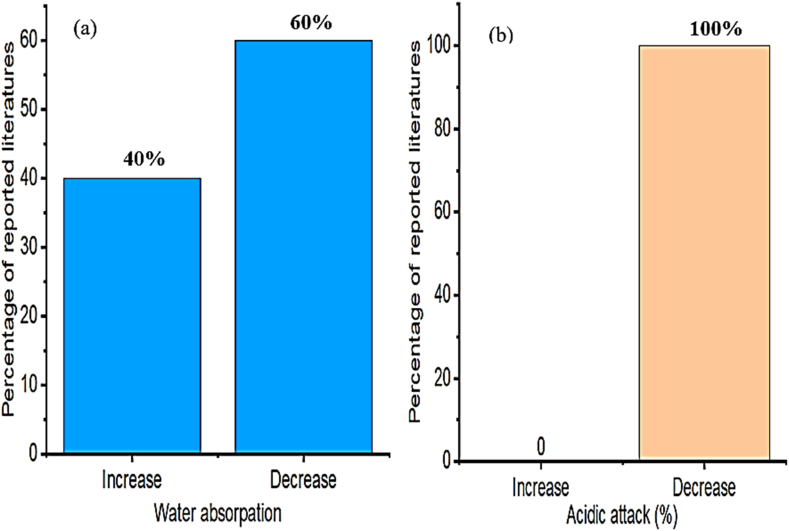
Summary from Table-6 that most researchers reported on the influence of zeolite on (a) water absorption and (b) acidic attack.

The replacement of cement with natural zeolite reduced the water absorption and chloride diffusion coefficient of concrete [125]. Besides this inclusion of zeolite in concrete increase sulfate resistance, and reduce water permeability compared to the reference concrete mixtures [128]. Hence as presented in Fig. 10a–b many researchers reported as adding zeolite in cementitious materials decreases water absorption and acidic attack, which is primarily due to the active pozzolanic reaction of zeolite in the hydration process. This reduction of water absorption is one of the factors that indicate improvement in durability [11]. A similar observation with Samimi et al. [258] investigated the influence of zeolite on compressive strength and chloride ion resistance. And found as employing zeolite in concrete ultimately enhances electrical resistivity, hence significantly improves the durability of concrete by reducing chloride ion migration.

**Fig. 11:**
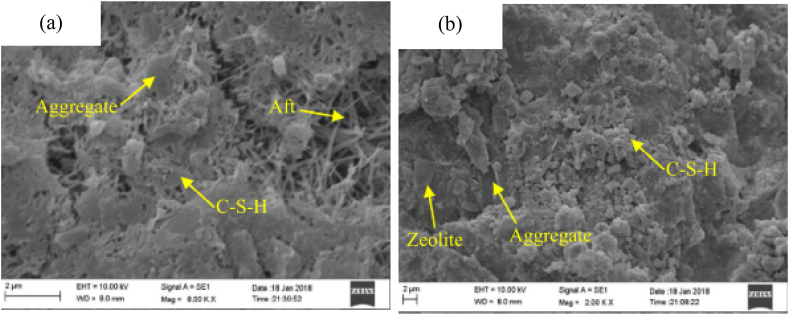
Microstructure of cement composite materials (a) with OPC and (b) OPC with zeolite by Ref. [261] permission Elsevier.

Replacement of zeolite to cement ratio is beneficial in enhancing crack width control ability and reduces shrinkage of cementitious materials [259]. As presented in Fig. 11a can observe from cementitious material without zeolite many pores, cracks, and low dense even hair-like structures; which can reduce the durability of the cementing materials by allowing penetration of water and acids, but as shown in Fig. 11b, the sample with zeolite can not seen such hair-like structures and many pores. Hence employing zeolite can reduce the occurrence of cracks, make dense microstructure and reduce pores. This is because of the pozzolanic reaction of zeolite with free calcium hydroxide and form additional *C*–S–H gel that is responsible for making denser microstructure and improve the durability of cementitious materials [260].

## 3 Artificial supplementary materials

### 3.1 Fly-ash

Fly ash is a coal-fired by-product from thermal power plants [262,263], mainly consisting of a high proportion of aluminum phase and predominantly spherical particles, which enhances the fluidity of the freshly mixed cement paste [260]. Also, the spherical shape of fly ash increases the volume of the structure due to the lower density of fly ash particles [229,264], and lessens the development of hydration products [265].

Raghav et al. [158] reported as fly ash needs activation due to two factors. First, the glassy surface layer of glass beads is dense, chemically stable, and preserves the inside constituents, which are porous, spongy, and amorphous. Second, the silica–alumina glassy chain of high Si, Al, and low Ca is stable; the chain must be decomposed to actively react with free lime that exists in cement. Consumption of free lime increases with increasing the fineness of fly ash [266]. So mechanical grinding, thermal activation, and chemical activation accelerate. Thus the significant level of replacement of Portland cement by fly ash is not only advantageous to the concrete but also minimizes the consumption of cement, and thereby decreases the effect of greenhouse gas [267,268].

Also, the replacement of fly ash in ordinary Portland cement with partial replacement has a positive effect on the mechanical and durability properties of cement composites [265,269,270], this is more similar to the result of most literature presented in Table 7, employing 5–30% of fly ash increase strength of cementitious materials while at higher replacement ratios negatively affect; moreover, the size of fly ash alters the properties of composites.

**Table 7:** Effect of fly ash on some of the mechanical properties and durability recorded by different researchers.

Dose range	W/C	Curing time	Optimum compressive strength		Optimum split tensile strength		Optimum flexural strength		Durability of addingfly ash comparing with control mixtures		References
			Dose (%)	Age (days)	Dose (%)	Age (days)	Dose (%)	Age (days)	Water absorption	Acidic attack	
0,5,10,15,20	–	28,60,90	10	28,60,90	–	–	–	–	–	Decrease	[271]
0,10,20,30	0.45	7,28	30	7,28	–	–	–	–	–	–	[272]
0,10,20,30,40,50	–	7,14, 21,28	10	7,14,21, 28	10	7,14, 21,28	–	–	–	–	[273]
0,10,30	0.45	3,7,28	10	3,7,28	10	7,28	10	7,28	–	–	[274]
0,15,30,45	0.55	3,7,14,28	30	3,7,14,28	30	3,7,14,28	–	–	–	–	[275]
0,10,20	0.34	7,28	10	7,28	10	7,28	10	7,28	–	–	[276]
0.5,10,15	0.50	7,28, 56,90	5	7,28,56, 90	5	7,28, 56,90	–	–	–	–	[10]
0,10,30,50,70	0.30	28,56	30	28,56	30	28,56	–	–	Decrease	Decrease	[277]
0,5,10,15,20,25	0.50	7,28, 90, 180,	10	7,28,90, 180,	10	7,28, 90, 180,	–	–	–	–	[278]
0,5,10	0.65	28,100,130	5	100,130	–	–	–	–	Decrease	Increase	[279]

**Fig. 12:**
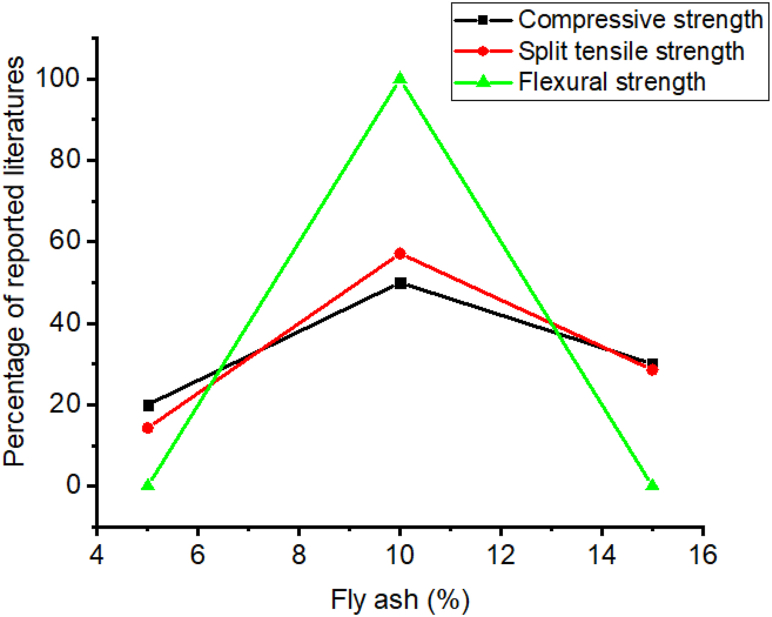
Summary from Table-7 that most researchers reported on the influence of fly ash doses for optimum compressive strength, split tensile strength, and flexural strength.

**Fig. 13:**
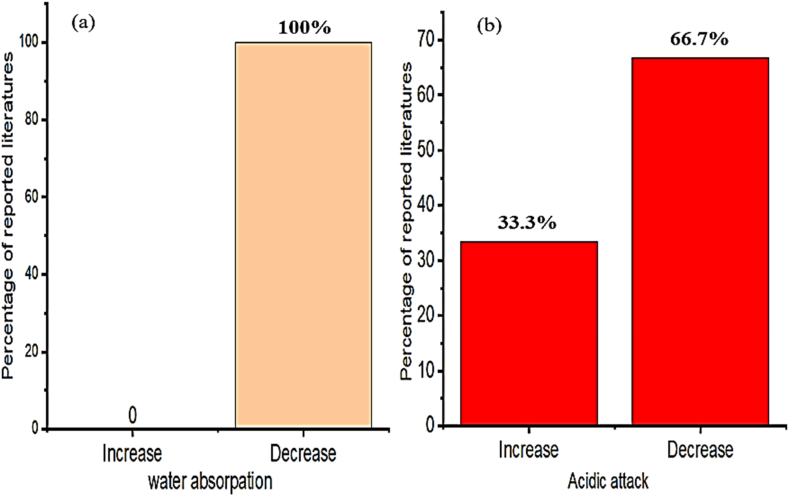
Summary from Table-7 that most researchers reported on the influence of fly ash on (a) water absorption and (b) acidic attack.

Also, the inclusion of fly ash improves the workability, durability, splitting tensile strength, and compressive strength of cementitious materials [74,274,[280], [281], [282], [283], [284]]. Specifically, as most literature studied shown in Fig. 12 addition of 10% of fly ash significantly improves the compressive, split tensile, and flexural strength of cementitious materials. Also as shown in Fig. 13a–b employment of fly ash highly reduce water absorption and acidic attack of cementitious materials compared with plane one. This is due to the fly ash micro filling ability reduces the pores and increase the density of cementitious materials which can protect the penetration of water and acids into the cement structural matrix [81,162,285].

### 3.2 Biomass ash

Sugarcane Bagasse Ash is biomass ash and a by-product of making juice from sugar cane by crushing the stalks of the plants [13], that can be considered as pozzolanic material and potentially can lessen free Ca(OH)_2_ in a cement matrix, which is owing to the pozzolanic reaction. Rice husk ash is another biomass ash that is highly reactive pozzolanic material from agro-waste by the combustion of rice husk [264,286].

As presented in Table 8 employing biomass ash improves strength and durability, especially many researchers reported using 10–20% biomass ash in cement composite can give optimum strength. That is due to the pozzolanic reaction of biomass ash favors the formation of secondary *C*–S–H gel and due to the amorphous state of reactive silica which can significantly enhance the strength and durability of concrete [58,158,195,[287], [288], [289], [290], [291], [292], [293], [294], [295]].

**Table 8:** Effect of biomass ash on some of the mechanical properties and durability recorded by different researchers.

Dose range	W/C	Curing time	Optimum compressive strength		Optimum split tensile strength		Optimum flexural strength		Durability of adding biomass ash comparing with control mixtures		References
			Dose (%)	Age (days)	Dose (%)	Age (days)	Dose (%)	Age (days)	Water absorption	Acidic attack	
0,5,10, 15,20	0.40	7,28, 90	10	28,90	–	–	–	–	Decrease	–	[295]
0,10	0.60	7,14, 28,56	10	7,14,28,56	10	7,14,28, 56	10	7,14, 28,56	–	–	[296]
0,10	0.60	7,14, 28,56	10	7,14,28,56	10	7,14,28, 56	–	–	–	–	[296]
0,10,20,30, 40,50	0.35	7,30, 60,90, 120, 150, 180	10	30,60, 90,120, 150,180	–	–	–	–	–	–	[297]
0,5,10, 15,20	0.55	7,14, 28	10	7,14,28	15	7,28	10	7,28	–	–	[298]
0,2.5,5, 7.5,10, 12.5, 15	0.60	3,7,28,90	10	7,28,90	10	7,28,90	10	7,28, 90	–	Decrease	[299]
0,5,10, 15,20	0.45	7,14, 28	10	7,14,28	10	7,14,28	–	–	–	–	[300]
0,5,10, 15,20	0.50	7,28	10	7,28	–	–	–	–	–	–	[141]
0,5,10, 15,20	0.45	7,14, 28,60	20	7,14,28,60	10	7,14,28, 60	–	–	Increase	Increase	[301]
0,5,10, 15,20,25	0.49	7,28, 90,180	10	7,28,90,180	10	28	–	–	–	Decrease	[143]
0,5,10, 15,20	0.50	7,28, 56	10	7,28,56	–	–	–	–	Decrease	Decrease	[302]

**Fig. 14:**
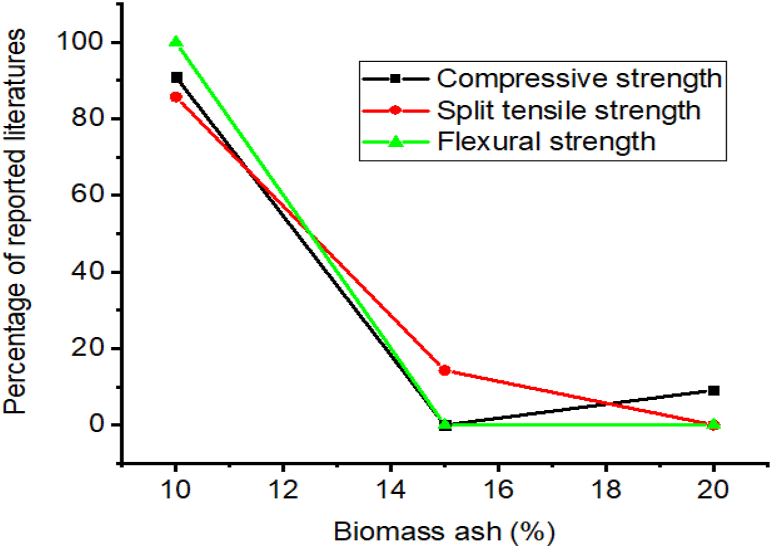
Summary from Table-8 that most researchers reported on the influence of biomass ash doses for optimum compressive strength, split tensile strength, and flexural strength.

Employing biomass ash mostly improves the compressive and splitting tensile strength of concrete compared to concrete without biomass ash [294,296,298,[303], [304], [305]]. Mostly as much of the literature reported shown in Fig. 14, 10% of biomass ash significantly improves the compressive, splitting tensile, and flexural strength of cement composites.

**Fig. 15:**
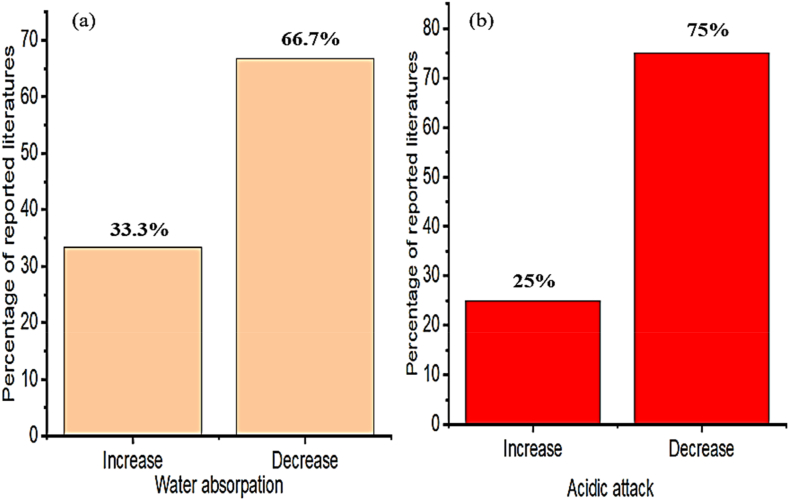
Summary from Table-8 that most researchers reported on the influence of biomass ash on (a) water absorption and (b) acidic attack.

As presented in Fig. 15a and b, employing biomass ash reduce water absorption and acidic attack of cementitious materials, specifically, rice husk ash in concrete reduces water absorption and chloride ion migration to the concrete [306]. That is because nano-silica in rice husk ash improves the formation of higher hydration products which fills the pores and makes denser microstructure which can reduce water absorption of concrete [51,136,307]. Also, the enhancement of durability of cementitious material reflected because of biomass ash from rice husk greatly can resist the migration of chloride ions, which means in another direction reduces the occurrence of corrosion in reinforced concrete [299].

Furthermore, Raghav et al. [158] reported as the addition of biomass ash decreases the acid attack, chloride diffusion, and corrosion rate of embedded steel rebar, especially, the unreacted silica in sugarcane bagasse ash acts as a pore filler, which can decrease porosity and voids in the concrete. So the reduction of porosity increases the resistance to chloride penetration and reduces the corrosion rate of steel rebar. However due to fineness of sugarcane bagasse ash highly increases the amount as cement replacement leads to high water demand, which can reduce workability of cement composite materials. The same observation with Tayeh, Hadzima-Nyarko et al. [308] as employing biomass ash, especially the agricultural waste of olive waste ash improves acidic and alkaline attack compared to the control concrete mixture.

**Fig. 16:**
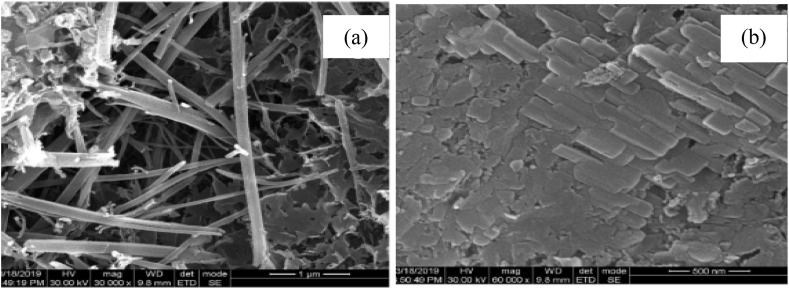
Microstructure of cement composite materials (a) with OPC and (b) OPC with biomass ash by Ref. [288] permission Elsevier.

Besides improving strength and durability, using biomass ash radically improve the microstructure of cement composites. As presented in Fig. 16a, cementing materials without biomass are not agglomerated structure though have many pores and stick-like structures that can allow the migration of water and acids which can reduce the durability of cementitious composite materials, however in Fig. 16b can observe cementitious materials with biomass ash which is highly dense microstructure, very fewer pores, and have uniformly structured matrix. This is very appreciable for the construction industry which requires high-performance of cementitious materials.

Generally, employing biomass ash from agricultural waste ash is beneficial for both technical and economical construction works. Hence it is mostly used for economic construction and for the reduction of environmental pollution [[309], [310], [311]].

### 3.3 Silica fume

**Fig. 17:**
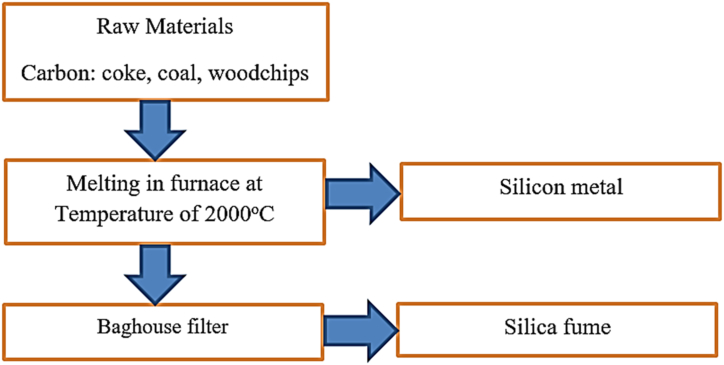
Schematic diagram of silica fume production by Ref. [313] permission Elsevier.

Silica fume is an artificial pozzolanic admixture [312] as shown in Fig. 17, the production process of silica fume is de-dusting from an electric furnace due to the manufacturing of silicon, zirconium, and ferrosilicon [38,147,[313], [314], [315], [316]]**.** Employing silica fume in cementitious materials reduces permeability which means increase durability, decreases workability, and improves compressive strength [74,147,313,[317], [318], [319]]. That is because silica fume particles are ultrafine and have a large surface area than cement particles which reduce workability by binding water in concrete/mortar, however, it refines the existence of pores and makes dense structure that improve durability and strength. In addition to this, since silica fume is waste from metal industries used in cement composite materials can lessen environmental pollution and construction costs.

**Fig. 18:**
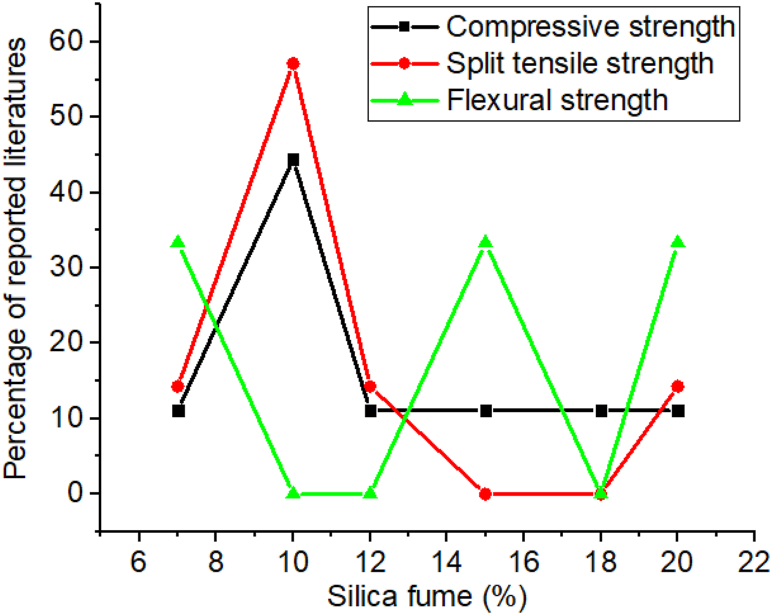
Summary from Table-9 that most researchers reported on the influence of silica fume doses for optimum compressive strength, split tensile strength, and flexural strength.

As presented in Table 9 many researchers found optimum strength and high durability by the addition of silica fume between 7 and 18% in cement composites, however, as shown in Fig. 18 most literature reported specific doses of silica fume of 10% by mass of cement which can give optimum compressive and split tensile strength. This mechanical property and durability of concrete improvement is due to the consumption of Ca(OH)_2_ by pozzolanic reaction and high pore refinement [156,[320], [321], [322], [323]]. Also increasing the contents of silica fume in cementitious material significantly reduces water absorption [148,324] and increases viscosity compared to the control mixtures [325]. This is due to the volume of void reduces by adding silica fume, because of the high level of specific surface area and high hydration reaction of silica fume [318]. Besides these, the inclusion of silica fume in cementitious material enhances the long-term corrosion resistance, and alkali-silica expansion, but also increases the carbonation depth in addition to refining the pores [314]. Moreover, recycling artificial SCMs from industrial waste conserves natural resource and prevent environmental pollution [326].

**Table 9:** Effect of silica fume on some of the mechanical properties and durability recorded by different researchers.

Dose range	W/C	Curing time	Optimum compressive strength		Optimum split tensile strength		Optimum flexural strength		Durability of adding Silica fume comparing with control mixtures		References
			Dose (%)	Age (days)	Dose (%)	Age (days)	Dose (%)	Age (days)	Water absorption	Acidic attack	
0,5,10,15,20	0.36	7,28	15	7,28	10	7,28	15	7,28	–	Decrease	[327]
0,5,10,15,20	0.32	7,28,56,90	10	7,28,56,90	10	7,28, 56,90	–	–	–	–	[312]
0,10,20,30, 40	0.55	3,7,14, 28	30	14,28	30	28	30	28	–	–	[328]
0,7,10	0.40	28	7	28	7	28	7	28	–	–	[329]
0,0.5, 1,5,10,15	–	3,7,14, 21,28	10	3,7,14, 21,28	–	–	–	–	–	–	[330]
0,2.5,5,7.5,10	0.48	28	10	0,2.5,5,7.5,10	10	28	–	–	–	–	[331]
0,6,12,18,24	0.48	3,7,28, 56	18	7,28,56	–	–	–	–	–	–	[332]
0,5,10	0.50	7,28,90	10	7,28,90	10	7,28, 90	–	–	Decrease	–	[333]
0,3,6,9,12,15	0.36	28	12	28	12	28	–	–	–	–	[334]

**Fig. 19:**
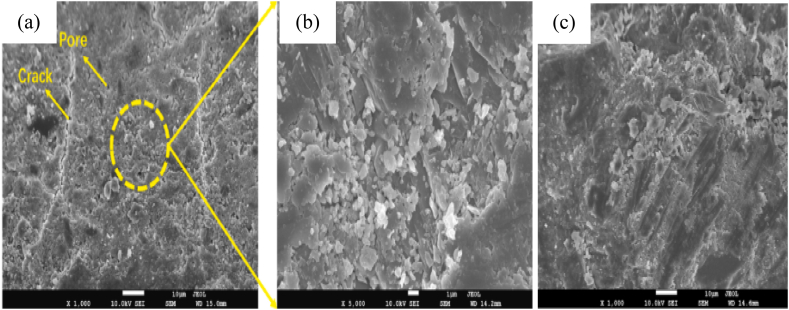
Microstructure of cement composite materials (a), (b) by OPC and (c) OPC with silica fume by Ref. [331] permission Elsevier.

As shown in Fig. 19(a–c), can observe cementitious composite materials without silica fume have many capillary pores and are not well-densed structures compared to the ones with silica fume. So the incorporation of silica fume can make dense microstructure and highly reduce pores which is more observable in the plane cementitious material. This is because of the active pozzolanic reaction of silica fume with free lime to form secondary calcium silicate hydrate gel which is responsible for changing the microstructures of cementitious material to be more denser than the one without silica fume [155,332].

Generally, from the review of different literature partial substitution of artificial and natural SCMs are beneficial for the improvement of physical, mechanical, durability, and microstructural properties of cement composite materials due to their micro-filling ability, having large surface area and by pozzolanic reaction between the free lime and SCMs to produce secondary calcium silicate hydrate (*C*–S–H) which can improve different properties of cement composite materials. However using of 100% OPC may lack all those important roles of SCMs, since the specific surface area cement is smaller than SCMs and free lime in OPC can not get more chances for the pozzolanic reaction to form additional *C*–S–H gel. In addition to this, using 100% OPC is costy and have an environmental problem, since its production requires much energy and releases more CO_2_ to the environment compared to composite cement. But standing on all these benefits of SCMs in cement composite materials, in the practical aspect of construction works can see adding of SCMs materials beyond the researchers result, which may loss all the importance of SCMs in cement composite materials.

## 4 Conclusions

The review of the various studies reported the beneficial effects of adding SCMs in cement composite, by improving mechanical properties, enhancing durability, and making dense microstructure of cement composite materials. The following conclusions are made based on the comprehensive review of this study.

• Partial replacement of bentonite, biomass ash, and kaolin isolately by 15% and volcanic ash by 20% in cement composite materials can give optimum compressive strength and splitting tensile strength.
• Addition of fly ash, silica fume, and zeolite isolately in cement composite materials by 10% reflected optimum compressive strength and split tensile strength.
• The review observed, as natural SCMs can more replace cement content to get the optimum strength of cement composite materials. This is an indication as natural SCMs can significantly reduce energy consumption and environmental pollution coming due to cement production by replacing cement more than artificial SCMs.
• Employing most of the artificial and natural SCMs significantly improve durability, especially reduce water absorption and improve acidic resistance compared to convectional cement composite materials. This is mainly due to most SCMs are fine and have a large surface area than cement particles which can fill the pores and reduce penetration of water and acids.
• Addition of SCMs in cement composite makes a dense microstructure of cement matrix. That is because, many of SCMs have micro-filling ability and actively react with free lime in cement to form extra *C*–S–H. This *C*–S–H potentially can participate in the improvement of the microstructural appearance and durability of the cement matrix. Hence, most of the reviewed literatures indicated that incorporation of SCMs in cement composite significantly improves durability, however silica fume and volcanic ash have seen very limited studies on water absorption and acidic attack resistance of blended cement composite. So the authors recommend future researchers focus on the effect of silica fume and volcanic ash on the durability of cementitious materials.

Generally, from most literatures have seen positive effects on the addition of artificial and natural SCMs in cement composite due to active pozzolanic reactions between reactive silica/alumina from SCMs and calcium hydroxide from cement that forms secondary calcium silicate hydrate (*C*–S–H) or calcium aluminate silicate hydrates (*C*-A-*S*-H) which can play a great role in the improvement of physical, mechanical, durability, and microstructural properties of cement composite materials.

## 5 Future perspectives

The most crucial consideration has been seen is that many of the reviewed literatures for the chemical composition of silica fume indicated extremely high silica content which is more than 90%, though ASTM C618 [86] recommends addition for the values of sulfur dioxide (SiO_2_), Aluminum oxide (Al_2_O_3_), and Iron oxide (Fe_2_O_3_) greater than 70%. So it is beneficial to isolately investigate the effect of sulfur dioxide in silica fume can possess on the mechanical, physical, and microstructural properties of cementitious materials. Also, deep investigation is necessary on the durability of silica fume composite cement materials since its observed very limited studies on water absorption and acidic attacks due to silica fume.

Generally, it is observed, as there are limited studies on volcanic ash employment on cement composite especially on researching its effect on flexural strength, split tensile strength acidic attack, and microstructure of construction materials, it is crucial to more analyze the effects of volcanic ash on mechanical properties, durability and microstructural change on cement composite.

## 6 Gap analysis of results and future recommendation

Although, all the reviewed literatures are important and solved the gap of knowledge concerning artificial and natural SCMs, but the following needs more considerations:

1. As presented in Fig. 20, more than 90% of reviewed literatures did not test the amount of chloride ions that exist in each of the sampled SCMs, however, knowing its content in every sampled SCMs is very crucial since can cause corrosion of steel bar in cement composite materials.

**Fig. 20:**
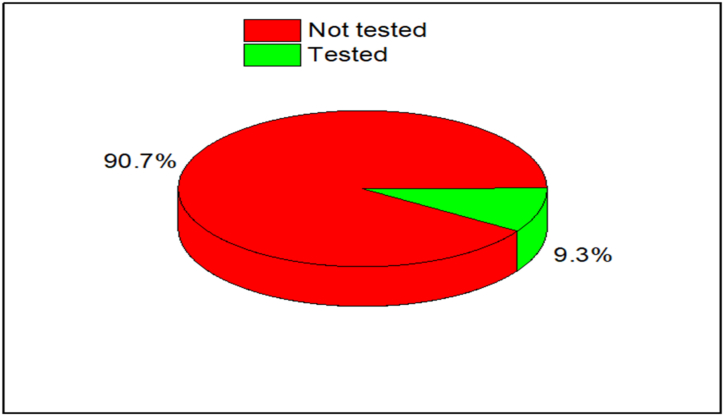
Summery from Table 1, Table 2, the percentage of chloride ion tests conducted on the reviewed literatures.
2. As shown in Fig. 21, all and more than half of the reviewed literatures conducted compressive and split tensile strength tests respectively. However, it is observed that more than 70% of the reviewed literatures did not conduct flexural strength, water absorption, and acidic attack tests. Especially, water absorption and acidic attack are very important indicators of the durability of cement composite materials employed with SCMs. In general, the review has seen very limited indicators of durability tests of cement composite materials like shrinkage, ultrasonic velocity, and toughness tests, hence the authors recommend the future studies focus more on the effect of artificial and natural SCMs on the durability of cement composite materials.

**Fig. 21:**
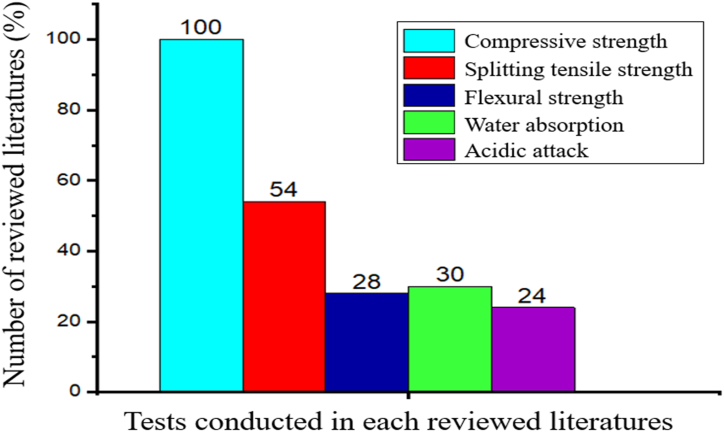
Summery from Table 3, Table 4, Table 5, Table 6, Table 7, Table 8, Table 9, the percentage of different tests conducted in each reviewed literatures.

## Author contribution statement

All authors listed have significantly contributed to the development and the writing of this article.

## Data availability statement

Data included in article/supp. Material/referenced in article.

## Declaration of competing interest

The authors declare that they have no known competing financial interests or personal relationships that could have appeared to influence the work reported in this paper.
